# Primary Progressive Aphasia: Use of Graphical Markers for an Early and Differential Diagnosis

**DOI:** 10.3390/brainsci11091198

**Published:** 2021-09-11

**Authors:** Alexandra Plonka, Aurélie Mouton, Joël Macoir, Thi-Mai Tran, Alexandre Derremaux, Philippe Robert, Valeria Manera, Auriane Gros

**Affiliations:** 1 Département d’Orthophonie de Nice, Faculté de Médecine, Université Côte d’Azur, 06000 Nice, France; mouton.a2@chu-nice.fr (A.M.); philippe.robert@unice.fr (P.R.); auriane.gros@univ-cotedazur.fr (A.G.); 2 Laboratoire CoBTeK (Cognition Behaviour Technology), Université Côte d’Azur, 06000 Nice, France; derreumaux.a2@chu-nice.fr (A.D.); valeria.manera@univ-cotedazur.fr (V.M.); 3 Institut NeuroMod, Université Côte d’Azur, 06902 Sophia-Antipolis, France; 4 Service Clinique Gériatrique du Cerveau et du Mouvement, CMRR, Centre Hospitalier Universitaire, 06000 Nice, France; 5 Département de Réadaptation, Faculté de Médecine, Université Laval, Québec, QC G1V 0A6, Canada; joel.macoir@rea.ulaval.ca; 6 Centre de Recherche CERVO (CERVO Brain Research Centre), Québec, QC G1J 2G3, Canada; 7 Laboratoire STL, UMR 8163, Département d‘Orthophonie, UFR3S, Université de Lille, 59000 Lille, France; thi-mai.tran@univ-lille.fr

**Keywords:** primary progressive aphasia, Alzheimer’s disease, graphical markers, graphical parameters, writing pressure, differential diagnosis

## Abstract

Primary progressive aphasia (PPA) brings together neurodegenerative pathologies whose main characteristic is to start with a progressive language disorder. PPA diagnosis is often delayed in non-specialised clinical settings. With the technologies’ development, new writing parameters can be extracted, such as the writing pressure on a touch pad. Despite some studies having highlighted differences between patients with typical Alzheimer’s disease (AD) and healthy controls, writing parameters in PPAs are understudied. The objective was to verify if the writing pressure in different linguistic and non-linguistic tasks can differentiate patients with PPA from patients with AD and healthy subjects. Patients with PPA (n = 32), patients with AD (n = 22) and healthy controls (n = 26) were included in this study. They performed a set of handwriting tasks on an iPad^®^ digital tablet, including linguistic, cognitive non-linguistic, and non-cognitive non-linguistic tasks. Average and maximum writing pressures were extracted for each task. We found significant differences in writing pressure, between healthy controls and patients with PPA, and between patients with PPA and AD. However, the classification of performances was dependent on the nature of the tasks. These results suggest that measuring writing pressure in graphical tasks may improve the early diagnosis of PPA, and the differential diagnosis between PPA and AD.

## 1. Introduction

Primary progressive aphasia (PPA) assembles a heterogeneous syndromic group of neurodegenerative pathologies characterised by a foreground and initially isolated language impairment that can later extend to cognitive functions such as computation, praxis, memory or executive functions [1,2,3]. It is a focal form of atrophy with great neuropathological heterogeneity, ranging from tauopathy to amyloidopathy or TDP-43 inclusions [4]. The prevalence of this disease is estimated at 3 per 100,000 [4], with a starting age assessed between 50 and 65 years [5] and a life expectancy of 10 to 15 years [6].

### 1.1. Diagnosis and Classification

PPA is diagnosed when three criteria overlap: (1) language is mainly damaged; (2) daily living activities are impaired during the initial stages of illness; and (3) word production and comprehension are impaired due to a progressive aphasic disorder and there is an underlying neurodegenerative disease [7]. This last criterion is still debated, based on the fact that PPA evolution from isolated language alteration to global cognitive impairment with multiple neuropsychiatric symptoms can lead to a change in diagnosis [8]. Additionally, language impairment that commonly lasts for about 6 years can represent the only symptom for 10 to 14 years, and is quickly impaired all along the degenerative process before being added to psychiatric and neurologic symptoms [9,10].

In 2011, a broad-ranging International Consensus Group published recommendations for the diagnosis and classification of PPA, establishing three different subtypes of this disease depending of the affected brain regions and the type of aphasic disorder [7]: the logopenic subtype (lvPPA), the non-fluent/agrammatic subtype (nfavPPA) and the semantic subtype (svPPA). A fourth subtype came to complete this classification: a mixed form or non-classified form [11].

lvPPA is defined by impaired word retrieval and phonologic errors that alter language fluidity. Sentence and word repetition are difficult due to a phonological loop disorder that also affects the understanding of long sentences with illness evolution [7,12,13]. lvPPA is characterised by a left posterior parietal or Perisylvian hypometabolism and an atrophy in the left posterior parietal lobe [12,14,15]. Studies have shown that AD is the most common underlying pathology of lvPPA [16].

nfavPPA is characterised by the presence of agrammatism in speech production, with impairments in understanding syntactically complex sentences [3,17,18]. Language production is laborious due to apraxia of speech with phonetic errors, although word comprehension is preserved. nfavPPA is related to dysfunctions in the frontal lobe, in Broca’s region, and the anterior parts of the insula [11,12,15,19]. Some studies have also exhibited parietal and temporal involvement [20]. Disorder of nfavPPA is most often frontotemporal lobar degeneration (FTLD) [16].

svPPA is characterised by the presence of a semantic language disorder with paraphasias in the expressive side and impairments in word comprehension in written or oral modalities, associated with a non-verbal semantic disorder [21,22]. svPPA’s anatomical lesions are located in the anterior temporal cortex and the inferior and middle temporal cortex [12,23,24,25]. An infiltration of several connecting beams passing through the temporal lobe have also been reported by fibre-tracking method (DTI) on a small sample (n = 5) [26]. As for nfavPPA, FTLD-type disorder changes are the most common in svPPA [16].

Mixed PPA is characterised by a combination of symptoms of the three main PPA variants with frequent impairment of word comprehension, apraxia of speech or agrammatism [27].

### 1.2. Early Diagnosis

Early diagnosis of PPA is important in clinical practice because its phenotype is complex, constantly evolving, and is crucial because it increases the possibilities of appropriate clinical interventions. In addition, diagnosis is complex: it has been shown that there is a delay of approximately 4 years between the onset of troubles and PPA diagnosis [6,28,29]. Moreover, the three PPA variants differ in terms of progression over time. lvPPA seems to follow the pattern of Alzheimer’s disease (AD) [30], which evolves to a generalised cognitive impairment, whereas other PPA types can be related to different diseases such as behavioural variants of FTLD, corticobasal degeneration or progressive supranuclear palsy [31].

Thus far, there has been no pharmacological treatment modifying or delaying PPA, but non-pharmacological interventions, such as speech therapy, have proven to be useful in compensating for and maintaining functional communications [32]. Early diagnosis is thus crucial to implement early and adapted interventions. Most of the scales available so far for PPA diagnosis are based on language production and comprehension in oral and written modalities. The main parameters assessed are performance (correct responses and mistakes) and response times. Writing disorders are also considered, such as dysorthography and, more specifically, spelling impairment [33,34], but no study has used graphical parameters such as writing pressure so far.

The use of new technologies allows more ecological and reproducible tests in comparison to certain scales or paper–pencil tests [35,36]. Computerised assessment batteries can build upon standardised and validated pencil-and-paper tests [37].

### 1.3. Contribution of Graphical Markers

With language symptoms being the earliest and most prominent signs in the early stages of the disease, graphical writing markers may constitute ecological markers of great interest for the early diagnosis of PPA [38].

Several studies have shown that graphic parameters are affected early in people with moderate to severe Alzheimer’s disease [38]. Studies have also shown that motor activity reveals language-related characteristics, due to the involvement of motor areas of the brain in writing, and that even mild disorders can be detected using motor parameters (reduction in written pressure) during language production tasks [39].

Handwriting requires the implementation of cognitive processes related to language as well as planning, coordination and motor execution. It has thus been shown that people with cognitive decline overall have a lower writing speed and pressure with a longer writing time, especially when analysing cursive loops [40]. Handwriting performance therefore exhibits significant changes, which it would be interesting to take into account within the framework of a classification of parameters characteristic of the neurodegenerative diseases such as AD, Parkinson’s disease (PD) or PPAs [41].

The use of a digital tablet with a stylus makes it possible to objectify the kinematic parameters of writing (pressure, stroke, velocity, jerk, and writing task time); therefore, this would allow a low-cost dissemination of this technology, especially if included in existing screening batteries [42].

The aim of this study was to confirm the initial findings of Gros et al. on a larger sample of PPA [41], concerning the role of writing pressure in differentiating PPA and controls, and to verify if writing pressure is also relevant to distinguish patients with PPA and Alzheimer’s disease.

## 2. Materials and Methods

### 2.1. Ethics

This study was approved by CPP Ile de France X (N° IDRCB: 2019-A00342-55 accepted on 11 September 2019). At the time of diagnosis, patients and relatives were informed of their inclusion in this study and could decline their participation or withdraw consent. Data were anonymised before the analyses.

### 2.2. Population

This was a prospective, multicentric study that included 5 French Neurology Departments (Nice, Angers, Nîmes, Saint-Brieuc, and La Rochelle). The patients were recruited from memory consultations in the various centres from June 2019 to February 2020. Eighty adults participated in this study, including patients with PPA (n = 32), patients with typical AD (n = 22) and healthy controls (HC) (n = 26) recruited in the memory centres. All the healthy controls were in good physical and mental health, reported no significant complaints related to cognition, and performed within the normal range on standardised neuropsychological tests. Only two patients (1 PPA, 1 HC) were left-handed. The demographic and clinical features of the three groups of participants are summarised in Table 1.

To be included in the study, the patients had to: be aged 40 years or more, have been diagnosed with PPA or AD according to the DSM-5TM criteria [42], have consulted in one of the investigation centres for cognitive, behavioural and/or motor difficulties, be able to read, write and speak French, benefit from social security coverage, and have no objection for inclusion on the study after reading the information note. The exclusion criteria for the patients and the healthy controls were the presence of a protective measure (guardianship or curatorship), a history of cerebrovascular disease, a history of psychiatric disorder according to the DSM-IVTR criteria [43], any neurological condition (except PPA and AD), traumatic brain injury, untreated medical or metabolic condition (e.g., diabetes, hypothyroidism) uncorrected hearing and vision problems, or prescribed medication with central nervous system sides effects likely to interfere with the carrying out of the tests.

Clinical data were reported retrospectively by the investigators and included: the etiological diagnosis of PPA, PPA variant according to Gorno-Tempini et al. criteria [7], the etiological diagnosis of AD according to the DSM-5TM criteria [42], the results of the various paraclinical examinations (cerebral MRI, PET-Scan, DAT scan, lumbar puncture), the current treatments, including the use of anticholinesterases or Memantine, the global level of cognitive functioning with the Mini Mental State Examination (MMSE), and the status of memory and language capabilities as well as their severity level.

### 2.3. Procedure

During the first visit, patients received explanations about the study and were given an information sheet. The investigator checked the inclusion criteria and signed a no-objection form. Various elements of the anamnesis were collected: age, gender, level of education, laterality, duration of the disease and familiarity or not with the touchpad devices.

When a patient was included in the study, the practitioner administrated the Detection Test of Language impairments in Adult (DTLA) and the tasks of graphic markers on an iPad^®^ tablet [43]. The DTLA test was chosen because of its accuracy for language disorders associated with neurodegenerative diseases. It is a standardised, rapid test, scored on 100 points, validated, and standardised in four French-speaking countries, as well as standardised according to 2 age groups and 2 levels of study. The DTLA test is composed of 9 subtests exploring the language functions most affected in neurodegenerative diseases, and its validation study showed that it has a good convergent validity, a good discriminant validity with healthy controls and a good test–retest fidelity.

### 2.4. Material and Variables

Graphical markers were collected on the written tasks of the DTLA with an Apple iPad ^®^ 2018 touchpad (model MR7F2NF/A) and an Apple Pencil ^®^ stylus model A1603. The stylus sample rate was 60 Hz, the screen accuracy was 1 pixel, and its resolution was 2048 × 1536. The application retrieved the position and tap pressure provided by the Apple stylus through the Safari browser. Pressure was measured as a percentage of the maximum pressure allowed by the stylus. These values were measured during plots, and updated every 17 ms.

**Figure 1:**
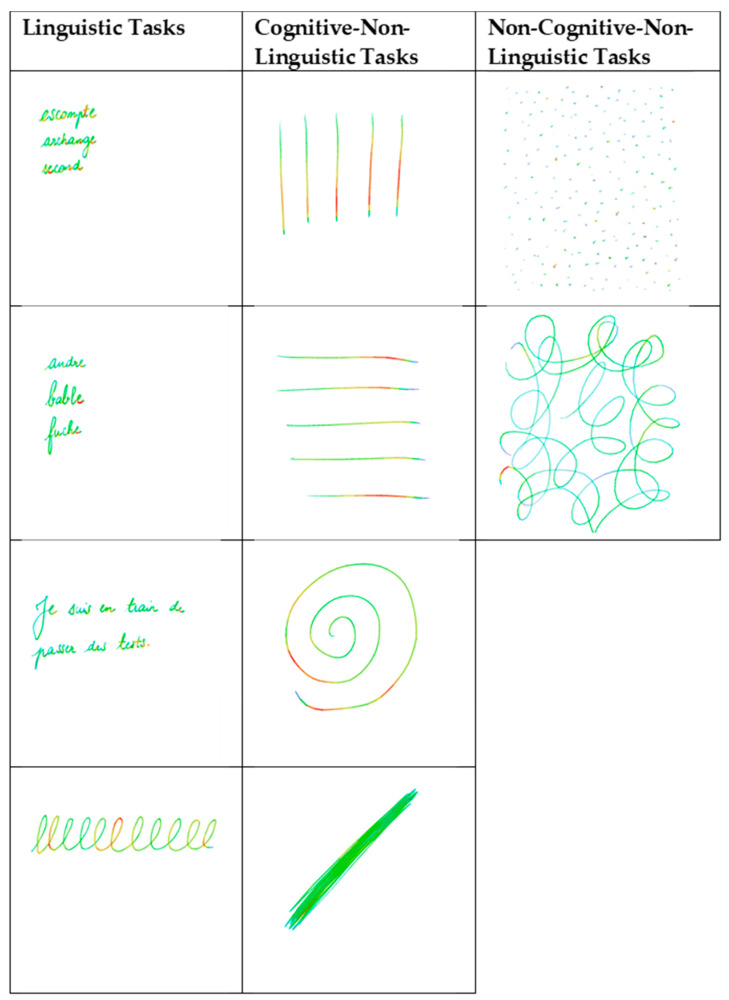
Graphical marker tasks. Linguistic tasks: words, nonsense words, sentence, letter ‘l’ loops. Cognitive non-linguistic tasks: vertical and horizontal lines, spiral, diagonals. Non-cognitive non-linguistic tasks: dots, filling loops. Writing pressure was collected on an iPad^®^ tablet. Red colour indicates the maximum pressure.

The following ten written tasks were analysed: four linguistic tasks, consisting of writing words to dictation, writing nonsense words to dictation, writing a spontaneous sentence, all part of the DTLA, and writing letter ‘l’ loops. Four cognitive non-linguistic tasks, consisting of writing vertical and horizontal lines, diagonals, and a spiral, and two non-cognitive non-linguistic tasks, consisting of writing dots and filling loops were performed. For the cognitive non-linguistic tasks of writing diagonals, the participants had 30 s to go back and forth as fast as possible between two squares presented on the screen. For the non-cognitive non-linguistic tasks, they had to fill the screen with dots and loops (Figure 1). For each task, we extracted the average (avgP) and the maximum (maxP) writing pressure, representing the pressure of the stylus on the screen (ranging from 0 to 1).

### 2.5. Statistical Analyses

Descriptive statistics were used to present demographic and clinical characteristics. Qualitative variables (sex) were presented using the frequency and percentage, and quantitative variables (age, years of education, MMSE score, and DTLA score) were presented using the mean and standard deviation (SD). The effects of the diagnostic group (PPA, AD and healthy controls) on quantitative demographic variables were tested using one-way ANOVAs for normally distributed variables (followed by LSD-corrected post hoc tests) and Kruskal–Wallis for non-normally distributed variables (followed by Bonferroni-corrected post hoc tests). The diagnostic groups differed in terms of mean age; therefore, we performed ANCOVAs on the average and maximum writing pressure using the diagnostic group (PPA, AD and healthy controls) as between-subject factor, and the age as a covariate (followed by LSD-corrected post hoc tests).

Qualitative variables (such as sex) were compared using the χ^2^ test. All statistical analyses were performed using IMB SPSS Statistics V20.0 software.

## 3. Results

### 3.1. Demographic and Clinical Information

Characteristics and clinical information of each group are reported in Table 1. No significant differences in gender (χ^2^_(2)_ = 5.03, *p* = 0.081) and the number of years of education (F_(2,77)_ = 0.31, *p* = 0.738) were found across the three groups. Age varied significantly across the groups (F_(2,77)_ = 6.34, *p* = 0.003). Specifically, post hoc LSD tests showed that participants in the control group were significantly younger than participants with PPA (*p* = 0.005) and AD (*p* = 0.002), whereas no difference between PPA and AD groups was found (*p* = 0.521). As expected, MMSE scores varied significantly across groups (F_(2,51)_ = 8.66, *p* = 0.001), with participants in the control group showing significantly higher MMSE scores than participants in the PPA (*p* = 0.001) and the AD (*p* < 0.001) groups. No difference between PPA and AD groups was found (*p* = 0.493). A significant difference in the results of the DTLA scale was found (H_(2)_ = 46.20, *p* < 0.001). Bonferroni-corrected post hoc tests revealed that participants in the control group had significantly higher DTLA scores than participants in the PPA (*p* < 0.001) and the AD (*p* < 0.001) groups. The difference between PPA and AD groups did not reach statistical significance (*p* = 0.838).

### 3.2. Graphical Markers

#### 3.2.1. Average Pressure (avgP)

Descriptive analyses (mean and standard deviation) for the average pressure in each task and for differences between linguistic and non-linguistic tasks are reported in Table 2.

The ANCOVA with Group as the between-subject factor and Age as a covariate revealed a significant effect of Group on avgP in the horizontal lines (cognitive non-linguistic) task (F_(2,41)_ = 3.26, *p* = 0.049). Specifically, paired post hoc comparisons (LSD-corrected) revealed that avgP was significantly higher in AD compared to controls (*p* = 0.035), and almost significantly higher in AD compared to PPA (*p* = 0.057). No significant effect of Group was found for the other tasks.

Concerning the differences between linguistic and non-linguistic tasks, a significant effect of Group was found on the difference between words and horizontal lines (F_(2,40)_ = 3.94, *p* = 0.027); specifically, subjects with AD showed a higher avgP in the horizontal lines compared to the words task, whereas the opposite was true for controls (*p* = 0.016) and PPA subjects (*p* = 0.049). The same pattern was also found for the difference between non-words and horizontal lines (F_(2,40)_ = 4.24, *p* = 0,021)—subjects with AD showed a higher avgP in the horizontal lines compared to the non-words task, whereas the opposite was true for controls (*p* = 0.016) and PPA subjects (*p* = 0.031)—and for the difference between horizontal lines and sentence tasks (F_(2,40)_ = 3.99, *p* = 0,026), with subjects with AD showing a higher avgP in the horizontal lines compared to the sentence task, whereas the opposite was true for controls (*p* = 0.032) and PPA subjects (*p* = 0.021). Finally, a significant effect of Group was found on the difference between letter ‘l’ loops (linguistic) task and (cognitive non-linguistic) diagonals task (F_(2,74)_ = 3.38, *p* = 0,039), with subjects with PPA showing a higher avgP in the diagonals compared to the cursive loops task, whereas the opposite was true for controls (*p* = 0,026) and AD subjects (*p* = 0,046). No other significant difference was found.

#### 3.2.2. Maximum Pressure (maxP)

Descriptive analyses (mean and standard deviation) for the average pressure in each task and for differences between linguistic and non-linguistic tasks are reported in Table 3.

The ANCOVA with Group as a between-subject factor and Age as a covariate revealed a significant effect of Group on maxP for the sentences (linguistic) task (F_(2,74)_ = 3.65, *p* = 0.031), with AD subjects showing a significantly higher maxP compared to the controls (*p* = 0.009). A significant effect of Group was also found for the horizontal lines (cognitive non-linguistic) task (F_(2,41)_ = 3.24, *p* = 0,049)—AD subjects showed a significantly higher maxP compared to the controls (*p* = 0.021)—and for the dots (non-cognitive non-linguistic) task (F_(2,74)_ = 4.12, *p* = 0,020), with subjects with PPA (*p* = 0.007) and AD (*p* = 0.032) showing a higher maxP compared to the controls. No significant effect of Group was found for the other tasks.

Concerning the differences between linguistic and non-linguistic tasks, a significant effect of group was found on the difference between letter ‘l’ loops and dots (F_(2,75)_ = 5.27, *p* = 0.007). Specifically, all subjects showed a higher maxP in the dots compared to the cursive loops task, but the difference was higher for PPA (*p* = 0.002) and AD subjects (*p* = 0.027) compared to the controls. Furthermore, an almost-significant effect of Group was found on the difference between letter ‘l’ loops and horizontal lines (F_(2,42)_ = 3.03, *p* = 0.059) with controls showing a higher maxP in the letter ‘l’ loops vs. the horizontal lines task, whereas the opposite was true for subjects with AD (*p* = 0.028). No other significant difference was found.

#### 3.2.3. Summary of the Main Differences between PPA and Healthy Controls

**Figure 2:**
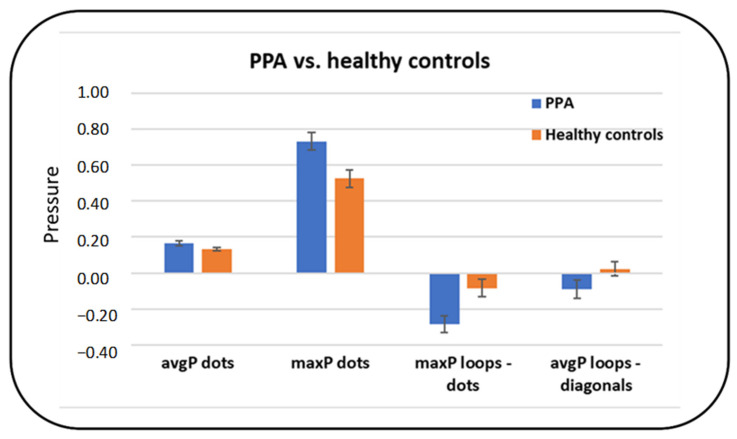
Differences in average and maximum writing pressure between patients with PPA and healthy controls.

Considering post hoc corrected comparisons, the most relevant tasks to distinguish PPA patients from healthy controls seemed to be the dots (non-cognitive non-linguistic) task and the letter ‘l’ loops (linguistic) task. Specifically, the maxP (*p* = 0,007) in the dots task was higher in PPA compared to healthy controls. Furthermore, the difference in maxP in the dots compared to the letter ‘l’ loops task was higher for PPA than for controls (*p* = 0.002). Finally, subjects with PPA had a higher avgP in the diagonals compared to the letter ‘l’ loops task, whereas the opposite was true for controls (*p* = 0.026) (Figure 2). 

#### 3.2.4. Summary of the Main Differences between PPA and AD

**Figure 3:**
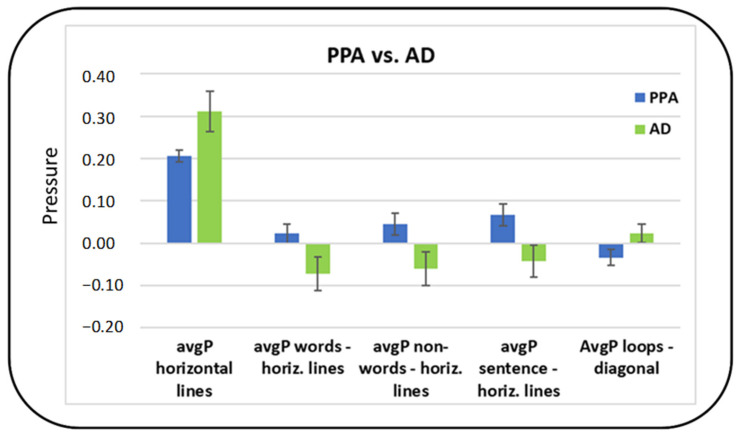
Differences in average and maximum writing pressure between patients with PPA and patients with AD.

Considering post hoc corrected comparisons, the most relevant feature distinguishing between PPA and AD patients was the avgP, whereas no significant differences were found for the maxP. In terms of tasks, the most relevant seemed to be the horizontal lines and diagonal lines (cognitive non-linguistic) tasks and the linguistic tasks. Indeed, differences in avgP were found for the horizontal lines task (AD>PPA, *p* = 0.057) and for the difference between horizontal lines and three linguistic tasks (words, non-words and sentence, *p* = 0.049, 0.031 and 0.021, respectively). Specifically, avgP in AD was higher in the cognitive non-linguistic tasks compared to the linguistic tasks, whereas avgP in PPA was higher in the linguistic tasks compared to the cognitive non-linguistic task. Finally, subjects with PPA showed a higher avgP in the diagonals task compared to the letter ‘l’ loops (linguistic) task, whereas the opposite was true for AD subjects (*p* = 0,046) (Figure 3).

## 4. Discussion

In the present study, we investigated the usefulness of graphical parameters collected in a handwriting protocol to differentiate patients with PPA from healthy controls, and patients with PPA from patients with AD. Significant differences in the average pressure and maximum pressure between PPA participants and healthy controls were found in the non-linguistic non-cognitive ‘dots’ task, and in the pressure difference between linguistic and non-linguistic ‘letter l loops’ and ‘dots’ tasks. These results show that PPA patients have a higher difference in the maximum pressure between a linguistic (‘letter l loops’) and a non-linguistic non-cognitive task (‘dots) than healthy controls. A previous study already showed that motor activity reveals language-related characteristics, due to the involvement of motor areas of the brain in writing [39]. This suggests that motor performance involved in linguistic and non-linguistic tasks may change in the presence of language disorders.

Other studies have shown an overall lower writing pressure in people with cognitive decline associated with AD compared to healthy people [40], with a lower pressure in most cognitively deteriorated groups [44]. Our results suggest the opposite with PPA patients in whom writing maximum pressure was significatively higher compared to healthy controls in the non-cognitive ‘dots’ task. Two major processes enter in handwriting: language processes and motor processes. Thus, writing could experience variations in different tasks depending on which process is reached [45].

Differences in pressure between a non-linguistic task and a linguistic task may suggest a decrease in the activity of the motor cortex during the graphic act, associated with a linguistic task for PPA patients (with a smaller difference between both). These results may be explained by the need for recruiting more cognitive resources during a linguistic task than during a non-linguistic task for PPA participants. Indeed, non-linguistic areas of the brain are usually more preserved in PPA than linguistic areas. This interpretation must be confirmed by an EEG exploration during writing in linguistic and non-linguistic tasks. These results are in line with other studies that show a relationship between language and gesture processing and the partial overlap of their neural representations. Indeed, a study demonstrated that PPA patients showed significant deficits on gesture discrimination tasks clustered with linguistic tasks as word and nonsense-word repetition, and writing-to-dictation [46].

The last aim of this study was to verify if graphical parameters could differentiate participants with PPA from participants with AD. Several studies have analysed graphical markers in patients with AD, but none in PPAs. Indeed, studies on PPAs focused only on the content of language in writing, and not on the graphic parameters. Thus, studies have shown letter insertion errors in patients with PPA, whereas they were absent in AD and mild cognitive impairment (MCI) patients, and that patients with PPA use more verbs than patients with AD [47].

Although the symptoms of AD are more cognitive than motor, it has been shown that motor dysfunction quantified by kinematic handwriting analysis is significantly correlated with MMSE scores in AD [48], and that pressure is lower in more cognitively deteriorated groups [44]. Graphic parameters and variability in the performance of patients with AD have been explained by a degradation of the motor programming, resembling that of Huntington’s rather than Parkinson’s disease patients, and may reflect frontal rather than basal ganglia dysfunction [49]. Finally, these studies suggest that MCI is also characterised by motor dysfunction and that writing with accuracy constraints may help identify those at risk of AD [50]. According to these studies, these deficits in graphical parameters seem to be more related to a motor dysfunction than a language impairment. Indeed, it has already been shown that in the mild phase of AD, lexico-semantic problems in the speaking process are possible but not predominant [51]. Thus, graphical markers in patients with AD seem more related to a deterioration in fine motor control and coordination [52,53].

Indeed, graphical markers seem to reflect the type of specific disorders in different pathologies and permit better comprehension of the nature of these deficits. In the same way, we have recently demonstrated a reduction in pressure, particularly in graphical activities, which have a spatial component in posterior cortical atrophy [54]. This result of a writing pressure change depending on the graphical task performed is in line with the results of a previous study on AD, and can be explained by the difference type of impairment between these pathologies [55].

Inconsistently with the literature on writing in patients with AD, our results show a difference between patients with PPA and patients with AD, with a predominant impairment in linguistic tasks in AD. Indeed, significant differences between the two groups were found for the cognitive non-linguistic horizontal lines task and for the difference between horizontal lines and three linguistic tasks. The average pressure in AD was higher in the cognitive non-linguistic tasks compared to the linguistic tasks, whereas the average pressure in patients with PPA was higher in the linguistic tasks compared to the cognitive non-linguistic tasks.

Contrary to the literature, these results suggest that graphical markers are not only a sign of motor and coordination disorders, but also a sign of cognitive and, more specifically, language disorders. Indeed, our results may suggest that patients with AD, despite an overall cognitive impairment, have a higher cognitive load than patients with PPA in linguistic tasks. In the same way, patients with PPA seem to have a high cognitive load for linguistic tasks but also in cognitive tasks (dysexecutive impairment). These results are in line with other studies that show early dysexecutive symptoms in patients with PPA [56] and a severe language impairment in patients with AD [57].

In conclusion, graphical markers may allow the performance of an early and differential diagnosis of patients with PPA and patients with AD. Writing pressure comparisons between linguistic and cognitive non-linguistic tasks reveal a difference in pressure between patients with PPA and healthy controls and patients with PPA and patients with AD. Indeed, in patients with AD, although the cognitive impairment is global, language impairment appears as an important diagnosis marker, such as in patients with PPA.

Other graphical kinematic parameters such as writing velocity could also be of interest for the classification of different subtypes of PPAs, because of the different anatomical pathways of degeneration. Thus, it has been shown that people with cognitive decline have a lower writing speed and pressure overall, with longer writing times [40]. However, to confirm these first results, a larger and more balanced PPA sample seems necessary.

Finally, this study highlights two main elements.

First, and on the scientific side, studying patients suffering from primary progressive aphasia, a clinical syndrome characterised by comparatively isolated language deficits, may provide direct evidence for anatomical and functional association between language deficits and gesture graphic particularity.

Second, on the clinical side, this study has shown the benefits of associating graphical markers to a rapid screening battery such as DTLA for the earlier and differential diagnosis of PPAs.

## Figures and Tables

**Table 1:** Demographic features of the groups of participants.

	lvPPA	nfavPPA	svPPA	AD	HC	*p*-Value
*N*	20	6	6	22	26	
*Female, n (%)* *	8 (40%)	3 (50%)	3 (50%)	9 (40.9%)	18 (69%)	0.081
*Age range (y)*	55–85	58–85	70–75	57–87	48–80	
*Mean age* **	73.1	69.5	71.3	73.6	65.7	0.003
*SD age* **	8.2	8.9	3.1	8.9	8.6	
*Mean Education (y)* **	10.1	11.5	9.8	10.1	11.1	0.738
*Education SD* **	3.8	3.7	5	4.8	5.4	
*Mean MMSE score* **	23.6	20.5	20.7	21.5	28.5	<0.001
*MMSE SD* **	5.4	4.2	5	4.9	1.7	
*Mean DTLA score* ***	74.5	44.6	58.3	74.8	95.9	<0.001
*DTLA SD* ***	16.6	10.9	19.9	17.1	5.6	

* χ^2^; ** *ANOVA*; *** *Kruskal–Wallis*, *p*-values refer to the overall comparisons between the three diagnostic groups (PPA, AD and HC).

**Table 2:** Average writing pressure in participants with PPA, AD and Healthy Controls.

	Task	Diagnosis	Mean	Standard Deviation
Linguistic Tasks	Words	PPA AD Controls	0.20 0.22 0.20	0.09 0.13 0.08
	Nonsense words	PPA AD Controls	0.22 0.23 0.21	0.10 0.14 0.09
	Sentence	PPA AD Controls	0.23 0.26 0.22	0.11 0.13 0.09
	Letter ‘l’ loops	PPA AD Controls	0.25 0.28 0.26	0.12 0.15 0.10
Cognitive Non-Linguistic Tasks	Diagonal	PPA AD Controls	0.28 0.26 0.24	0.14 0.13 0.10
	Vertical	PPA AD Controls	0.28 0.30 0.22	0.08 0.15 0.12
	Horizontal	PPA AD Controls	0.21 0.31 0.18	0.55 0.21 0.08
	Spiral	PPA AD Controls	0.25 0.26 0.25	0.12 0.11 0.09
Non-Cognitive Non-Linguistic Tasks	Dots	PPA AD Controls	0.17 0.19 0.13	0.07 0.09 0.04
	Filling Loops	PPA AD Controls	0.28 0.31 0.27	0.11 0.15 0.09

**Table 3:** Maximum writing pressure in participants with PPA, AD and Healthy Controls.

	Task	Diagnosis	Mean	Standard Deviation
Linguistic Tasks	Words	PPA AD Controls	0.61 0.66 0.55	0.30 0.26 0.23
	Nonsense words	PPA AD Controls	0.58 0.61 0.54	0.30 0.30 0.22
	Sentence	PPA AD Controls	0.65 0.78 0.53	0.33 0.27 0.25
	Letter ‘l’ loops	PPA AD Controls	0.45 0.48 0.44	0.23 0.22 0.23
Cognitive Non-Linguistic Tasks	Diagonal	PPA AD Controls	0.54 0.49 0.42	0.27 0.25 0.21
	Vertical	PPA AD Controls	0.55 0.57 0.43	0.19 0.23 0.21
	Horizontal	PPA AD Controls	0.45 0.58 0.32	0.21 0.30 0.16
	Spiral	PPA AD Controls	0.44 0.47 0.40	0.26 0.21 0.19
Non-Cognitive Non-Linguistic Tasks	Dots	PPA AD Controls	0.73 0.71 0.53	0.27 0.25 0.24
	Filling Loops	PPA AD Controls	0.52 0.54 0.51	0.20 0.22 0.20

## Data Availability

The data reported are part of an ongoing registration program. Deidentified participant data are not available for legal and ethical reasons. Anonymised data will be made available for research purposes, upon request and specifical approval of the database advisory board and ethical committee.
